# Genomic evidence supports the introgression between two sympatric stickleback species inhabiting the White Sea basin

**DOI:** 10.1016/j.heliyon.2021.e06160

**Published:** 2021-02-08

**Authors:** Artem Nedoluzhko, Fedor Sharko, Svetlana Tsygankova, Eugenia Boulygina, Amina Ibragimova, Anton Teslyuk, Jorge Galindo-Villegas, Sergey Rastorguev

**Affiliations:** a Faculty of Biosciences and Aquaculture, Nord University, 8049 Bodø, Norway; b Institute of Bioengineering, Research Center of Biotechnology of the Russian Academy of Sciences, 119071, Moscow, Russia; c National Research Center “Kurchatov Institute”, 123182 Moscow, Russia

**Keywords:** *Gasterosteus aculeatus*, Introgression, *Pungitius pungitius*, RAD-Seq, Stickleback, Transposable elements

## Abstract

Interspecies hybridization is driven by a complex interplay of factors where introgression plays an important role. In the present study, the transfer of genetic material, between two quite distant fish species from different genera, through spontaneous hybridization was documented with dedicated molecular and bioinformatics tools. We investigate the genomic landscape of putative stickleback-relative introgression by carefully analyzing the tractable transposable elements (TE) on the admixed genome of some individuals of two sympatric stickleback species inhabiting northwestern Russia, namely the three-spined (*Gasterosteus aculeatus*) and the nine-spined (*Pungitius pungitius*) sticklebacks. Our data revealed that unique TE amplification types exist, supporting our proposed hypothesis that infers on the interspecific introgression. By running a restriction site-associated DNA sequencing (RAD-Seq) with eight samples of *G. aculeatus* and *P. pungitius* and subjecting further the results to a contrasting analysis by variated bioinformatic tools, we identified the related introgression-linked markers. The admixture nature observed in a single sample of the nine-spined stickleback demonstrated the possible traces of remote introgression between these two species. Our work reveals the potential that introgression has on providing particular variants at a high-frequency speed while linking blocks of sequence with multiple functional mutations. However, even though our results are of significant interest, an increased number of samples displaying the introgression are required to further ascertain our conclusions.

## 1 Introduction

Interspecific hybridization in animals has been considered for a long time as an abnormal process leading to the destruction of reproductive isolation [1]. In the early 20th century, supporters of this viewpoint increased after the description of postzygotic isolation (PSI) [2]. The PSI supposes that the allele incompatibility increases as the square of the genetic distance between species complicate the hybridization between distant species. Nevertheless, while the interspecific hybridization is a natural event, it is also an active participant in some evolutionary process as the speciation or the new trait acquisition. In support of the same, it has been demonstrated that 25% of the plant species and more than 10% of animals from different taxa present traces of hybridization [3,4]. In contrast, the theoretical foundations of hybridogenic speciation propose the formation of reproductive barriers between hybrids and parents as the possible genetic mechanisms [5]. Fishes are not the exception, and thus the interspecific hybridization events have been appreciated as a quite common event among this phylogenetic group [6,7].

Nevertheless, the following logical question is placed forefront among the previously described concepts. Why, despite the existance of PSI, hybridization is still a present common feature in nature? In this regard, variated hypothesizes attempt to address the critical role of the hybridization in evolution. While still in debate, the most sounded hypothesis establishes a dramatic acceleration of the natural selection power by increasing the genetic variability resulting from the same [8,9,10]. However, studies demonstrate that hybridization allows offspring to obtain new traits [11,12,13], and conquer new habitats and ecological niches [8,9,10,14]. Such studies showed that hybridization is also part of the vertebrate speciation [5,15].

The high-throughput sequencing and genotyping era opened great avenues for studying in detail the hybridization involvement in the evolutionary processes [16]. To do it so, the species from the family Gasterosteidae (order *Gasterosteiformes*) are accessible elements to perform evolutionary studies, speciation, and several aspects of functional biology [17,18,19,20]. Notably, the three-spined stickleback (*Gasterosteus aculeatus*) is a well-studied model vertebrate species that acquired the freshwater adaptation due to the "freshwater adaptive standing genetic variation" – the quite extended genomic loci that significantly differ between marine and freshwater forms of this species [21,22]. The marine three-spined stickleback ancestor perhaps obtained these "divergence islands" from a close related freshwater species. Just recently, the hybridization between the marine and freshwater forms of the three-spined stickleback has been proved by using genetic and morphological data. Besides, an extended ecological potential in the hybrid offspring has been clearly described when comparing the hybrids with the parent forms [23,24,25]. Conversely, it is well recognized that some species from the genus *Pungitius*, like *P. pungitius* with *P. tymensis*, or *P. platygaster* with *P. pungitius* can form fertile interspecific hybrids with the ability to produce viable backcrosses [26,27]. However, according to recent literature, intergeneric hybrids of sticklebacks have not been described so far.

In the present research, we hypothesized the possible genomic introgression between two separate Gasterosteidae species belonging to two distinct genera, *Pungitius* and *Gasterosteus*. To validate our hypothesis, we first ascertain through the exhaustive analysis of several molecular pieces of evidence achieved by carefully analyzing of transposable elements (TE) and restriction site-associated DNA sequencing (RAD-seq) on a small number of specimens. Subsequently, the application of different bioinformatics approaches provided novel interspecies hybridization marks specific for the Gasterosteidae. Besides, our results also put forward a solid foundation on the use of transposable element analysis as a novel, low time consuming, and affordable method within several population-wide studies designed to find intergeneric genomic introgression [28]. In the meanwhile, we predict that further population genome-wide studies will shed light on this new hypothesis.

## 2 Results

**Figure 1:**
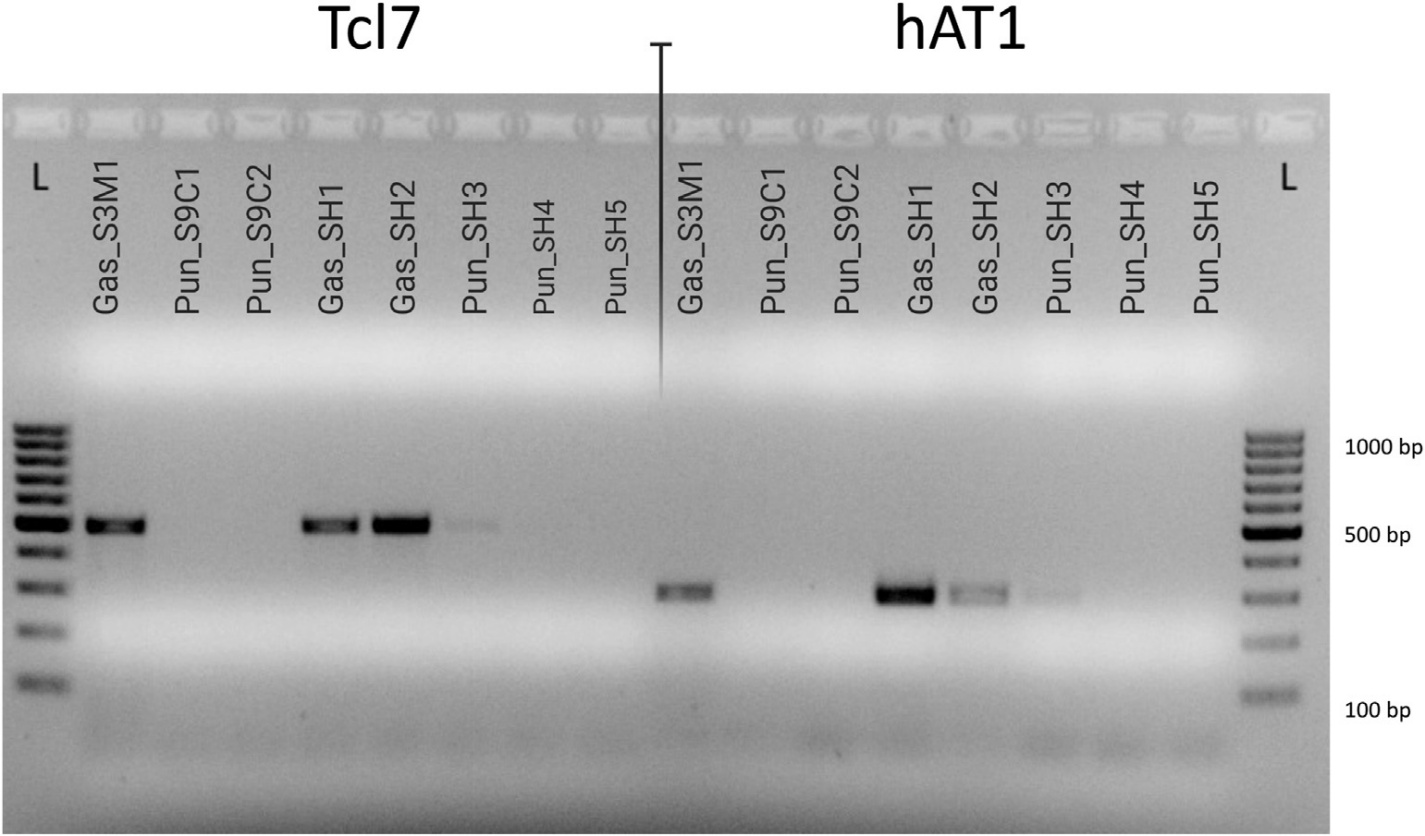
The example of gel electrophoresis for Tcl7 and hAT1 loci confirming the presence of three-spined transposable mobile elements (TE) in the nine-spined stickleback samples. L – GeneRuler 100 bp DNA Ladder (Thermo Fisher Scientific, USA).

A TE analysis was performed to determine the possible presence of introgressive hybridization between the three- and nine-spined stickleback species. DNA electrophoresis of the TE PCR products revealed that various types of elements are differentially amplified among the analyzed samples. However, in two loci, the Ltr89 and Tcl5, the TE were equally represented in both species' samples. For all the remaining loci, only the three-spined stickleback samples TE were mostly constant (Figure 1; Figure S1).

Strikingly, the SH3 sample, suspected as an admixed specimen of the nine-spined stickleback, produced a clear noticeable signal in most amplified profiles. Nevertheless, weaker than in the three-spined fish samples. Besides, some remaining samples of the nine-spined fish either show a weak signal at several loci (e.g., gyp13_10). Whatever the case, the fact that some nine-spined samples showed the presence of the three-spined like TE provided the required evidence for conducting further analysis focused on expanding the genomic introgression knowledge between the three- and nine-spined sticklebacks.

To gain further insights into the genomic signals of introgression in the Gasterosteidae family, using the Illumina platform, we performed a RAD-Seq analysis of the DNA isolated from the eight stickleback samples described below (see: Table 5). The number of Illumina reads produced and mapped for all samples are shown (Table 1).

**Table 1:** Illumina generated reads and mapping statisti2cs on obtained reads mapped to three-spined stickleback and European sea bass genomes.

Library	Species	SRA (Accession number)	Number of reads passed the QC filter	Number of reads mapped to *G. aculeatus* reference genome	Mapped reads (%)	Number of reads mapped to *D. labrax* reference genome	Mapped reads (%)
SH1	*G. aculeatus*	SRR8790928	13,761,328	13,070,263	94.98%	2,300,166	16.71%
SH2		SRR8790927	12,833,168	12,181,319	94.92%	2,150,430	16.76%
S3M1		SRR8790929	15,830,072	14,214,295	89.79%	2,533,393	16.00%
SH3	*P. pungitius*	SRR8790926	8,979,744	5,660,455	63.04%	1,663,250	18.52%
SH4		SRR8790925	11,431,390	6,820,235	59.66%	1,842,669	16.12%
SH5		SRR8790924	13,188,552	8,168,945	61.94%	2,136,397	16.20%
S9C1		SRR8790923	11,009,768	6,955,650	63.18%	1,997,330	18.14%
S9C2		SRR8790922	11,008,146	6,865,711	62.37%	1,906,997	17.32%

**Figure 2:**
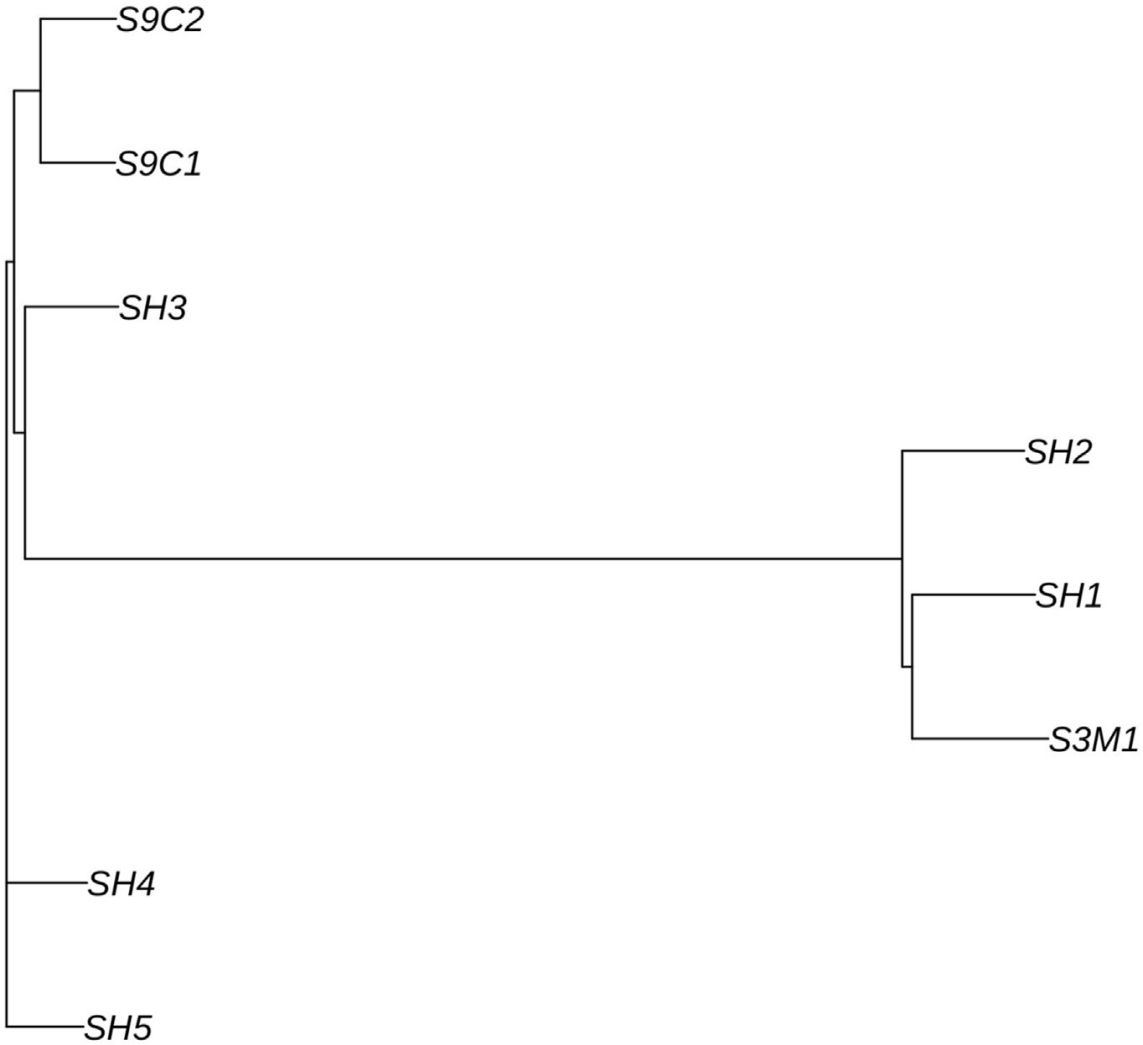
Cluster analysis for the three- and nine-spined stickleback samples, based on Euclidean distances between their genotypes.

Resulting from mapping the RAD-Seq data to the dicLab v1.0c reference genome, 108,969 loci in all samples were determined. However, after converting the vcf files to the genlight (adegenet) format, the total loci number decreased by 4,062. The reduction observed in the loci number resulting from the conversion is attributed to the particular characteristic of the genlight format that does not support loci with more than two alleles. We then created a distance matrix for all samples based on their genotypes' dissimilarity and conducted a cluster analysis based on the Neighbor-Joining method (Figure 2).

The genotypes defined in the vcf file were used for the admixture analysis in the Structure tool [29]. The Structure analysis of the nine-spined stickleback SH3 sample confirmed our previous assumption of this specimen's admixture origin. Besides, the SH2 sample of the three-spined stickleback specimen, collected in the same location as the SH3 sample, also displayed traces of intergeneric hybridization in its genome. However, despite the source, the admixture value present in both cases is less than one percent, which is extremely low but in a large loci number, supporting the reliability of the present result.

Based on the structure analysis, the inferred ancestry of individuals in the SH2 and SH3 libraries obtained from the three- and nine-spined sticklebacks, respectively share contrasting clusters on the 0.7 % level (Table 2). The quantitative values of an admixture could be revised because only a part of the whole genomes was analyzed. Moreover, we used only the available reference genomes from distant species, but the possibility of intergeneric genomic introgression is clearly inferred.

**Table 2:** Inferred ancestry of individuals based on the Structure analysis.

Library	Species	Inferred clusters		90% probability interval	
SH1	*G. aculeatus*	1.000	0.000	0.999,1.000	0.000,0.001
SH2		0.993	0.007	0.991,0.995	0.005,0.009
S3M1		1.000	0.000	0.999,1.000	0.000,0.001
SH3	*P.pungitius*	0.007	0.993	0.005,0.008	0.992,0.995
SH4		0.000	1.000	0.000,0.001	0.999,1.000
SH5		0.000	1.000	0.000,0.001	0.999,1.000
S9C1		0.000	1.000	0.000,0.001	0.999,1.000
S9C2		0.000	1.000	0.000,0.001	0.999,1.000

We selected three-spined - S3M1 and nine-spined - S9C1 and S9C2 specimens as test samples, and all other nine-spined specimens compared iteratively against them. D-statistics value increased for SH3 sample (Table 3). The value of 0.32 and 0.34 for S9C1 and S9C2 respectively are positive, which means there was an allelic frequency shift in the direction of convergence of the nine-spined SH3 sample with an S3M1 three-spined sample. Z-score of 3.9 means that the confidence of the admixture is high [30]. The same results we obtained when other three-spined and nine-spined specimens, accordingly, were used as test samples (data not shown).

**Table 3:** Results of genomic introgression test using the European sea bass (*Dicentrarchus labrax*) as outgroup.

Outgroup	Population 1	Population 2	Population 3	D-stat	Z-score	ABBA	BABA	Number of SNPs
*D.labrax*	S3M1	S9C1	SH3	0.3258	3.899	420	220	10,044
*D.labrax*	S3M1	S9C1	SH4	0.0223	0.276	270	260	10,044
*D.labrax*	S3M1	S9C1	SH5	0.0774	0.8	280	240	10,044
*D.labrax*	S3M1	S9C2	SH3	0.346	4.916	440	210	10,044
*D.labrax*	S3M1	S9C2	SH4	0.0542	0.707	260	230	10,044
*D.labrax*	S3M1	S9C2	SH5	0.1137	1.251	280	220	10,044

**Figure 3:**
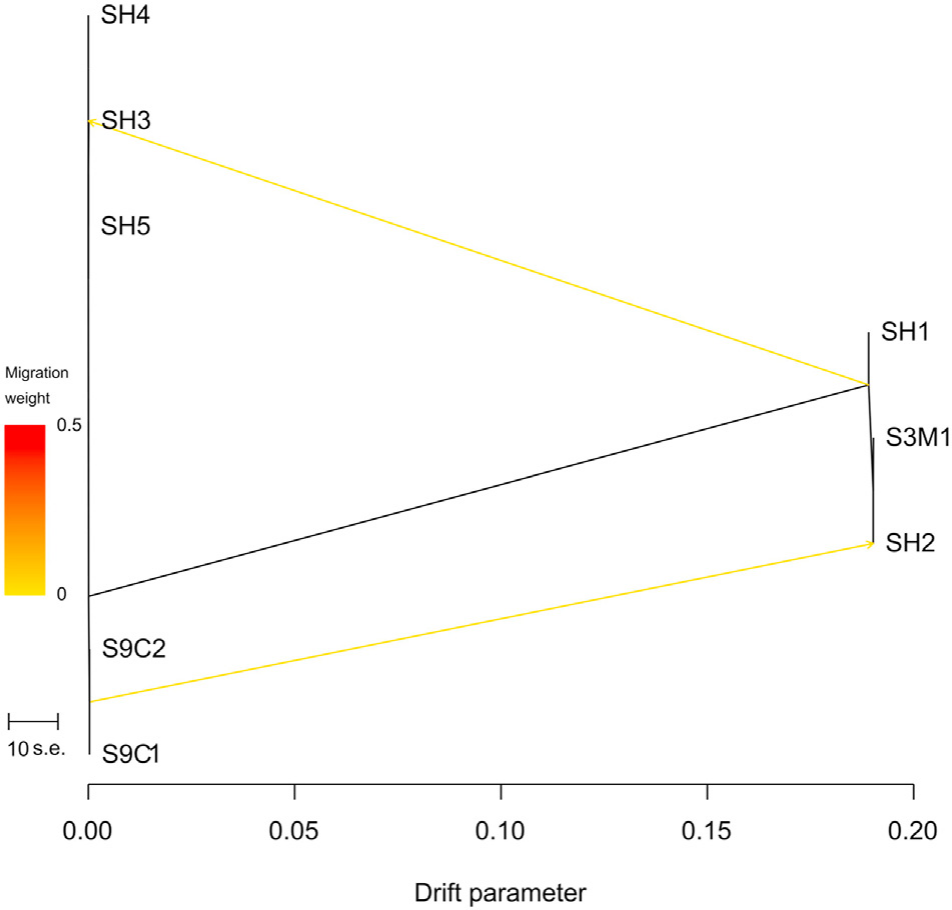
The evidence for admixture in the two stickleback species obtained by the TreeMix software analysis.

We conducted an additional introgression test based on ancestry graph models, implemented in the TreeMix software package by using a large number of SNPs to estimate the historical relationships among populations. The migration events in the TreeMix test splits and goes from the root node of the three-spined subgraph to the nine-spined sample (SH3) as well as from the nine-spined subgraph to the three-spined sample (SH2) (Figure 3). Simultaneously, the latter result with SH2 specimen was not supported by Structure and D-statistics analyses.

Despite that each specimen was collected, carefully stored on individual tubes, and followed a strict procedure on processing the DNA library, a remote possibility of DNA contamination among samples of the three- and nine-spined specimens exist. At first glance, this fact could explain the unexpected admixture results. To exclude the possibility that a sample has been mixed and sequenced, an assessment of mapped data for mismatches was conducted. The results revealed that SH3 admixed sample does not differ significantly from the rest of the samples. Even more, the SH3 admixture has the smallest mismatch rate comparing other specimens (Table 4).

**Table 4:** Test for contamination. Relative mismatch quantity in mapping data of three-spined and nine-spined stickleback specimens.

Specimen	S3M1	S9C1	S9C2	SH1	SH2	SH3	SH4	SH5
Mismatch quantity	0.07941	0.07559	0.07548	0.08059	0.07905	0.7356	0.07691	0.07621

Therefore, these results expressing the relative mismatch quantity in mapping data, sufficiently support the exclusion on the possible admixed sample contamination.

## 3 Discussion

Hybridization between evolutionary distant animal species has been traditionally perceived as a rare event in nature [31,32,33]. However, recent studies propose the introgressive hybridization as a widespread phenomenon among closely related vertebrate species [34,35]. Interestingly, this feature is particularly frequent among teleost fish [36,37]. Nevertheless, this effect is quite probably related only due to the total number of fishes exceeding by far that of all other vertebrates. Whatever the case, the new insights gained from different models and non-models teleost species and sequencing projects have recently revealed several peculiar features of fish genomes that might have played a particular role in fish evolution and speciation [38].

In this study, our efforts were focused on providing a multilevel survey on the evaluation of a possible introgression between the three-spined and nine-spined stickleback species. For this purpose, different powerful bioinformatical methods which have shown previous success on linking loci and correlating allele frequencies [29], revealing admixture processes [30], and inferring population splits and mixtures from genome-wide allele frequency data [39], were approached.

Besides, to increase the data accuracy, we developed a TE PCR-based identification system that allows for finding traces of previous introgression events between diverse stickleback species. The introgression process usually induces the burst of TE in the resulting hybrids [28]. Therefore, it is assumed that TE play essential roles in animal evolution, suggesting this part of the genome as a very sensitive marker of introgression. In our study, resulting from the emerged TE PCR products, and following an electrophoretic approach, we gave light on the apparent variation on the differential amplification of the TE between the two distant sympatric species of *Gasterosteiformes*, the three- and nine-spined sticklebacks.

The interspecies hybridization was expected to increase the TE activity in their hybrid offspring. In agreement with our findings, it has been previously shown that interspecific hybridization disrupts the stability of this part of the genome resulting in mutations and genetic instability [40]. We found that a single nine-spined (SH3) stickleback sample, presenting a reduction in the number of spines, showed TE specific to the three-spined stickleback (particularly, Tcl7, hAT1, hAT3, and Gyp9_4). The emerging evidence revealed that our novel approach succeeds in providing the required evidence on TE-elements' usage as an excellent molecular marker for introgression detection in fish. Thus, as a highlight of the present study, we introduce a novel TE PCR approach that provides fast and accurate evidence of the admixture process between species from the different genera - *G. aculeatus* and *P. pungitius*.

RAD-Seq has been proposed as the tool of choice in introgressive hybridization studies across individuals of non-model organisms [41,42]. For example, using RAD-Seq a secondary hybridization event at the end of the last glaciation period between two sole African species, *Solea senegalensis*, and *S. aegyptiaca*, has been reported despite a lengthily allopatric separation exists [43]. Furthermore, using the same tool, the genetic admixture and interspecies hybridization between different cichlid species from the *Alcolapia* genus inhabiting the African lake Natron has been recorded [44]. Together, these examples emphasize the importance of the RAD-seq analysis for interpreting hybridization patterns. In the present study, the RAD-Seq analysis revealed a strong relationship among the adaptive allelic character sets of the freshwater nine-spined (*P. pungitius*) stickleback acquired from its three-spined marine relative during the introgression process.

Note that here we did not use a well-annotated *G. aculeatus* genome. Instead, the European sea bass genome sequence served as reference, avoiding in this way the possible bias in the mapping efficiency. Besides, several approaches, including an assessment of mapped data for mismatches, and contamination test, were followed to increase the confidence in the results obtained from a limited number of specimens in this proof-of-concept investigation. So, thus far, the comparative bioinformatical analysis based on Structure, Treemix, and D-statistics methods of genomic data for this and other three-spined and nine-spined stickleback samples provides a definite milestone on the suspected admixture origin of SH3 specimen. The possibility of such hybridization events was explored toward the interspecific level [45]. However, only a few but sporadic findings showed the examples of intergeneric hybridization [37,46]. Thus, our results suggested that introgressive hybridization between the three- and nine-spined stickleback species adds a new case example of intergeneric hybridization.

Besides, we inferred that the newly TE-based PCR analysis should become a convenient type of preliminary experiment in case of in-depth screening of the populations where interspecies and intergeneric hybridization are expected. Whatever the case, we anticipate that further population-wide genomic studies will shed light on the adaptivity hypothesis of intergeneric hybridization between Gasterosteiformes.

Interestingly, along with the molecular findings, the ecological characteristics of the inhabiting aquatic niches of both stickleback species either implies a strong challenge to overcome. While the three-spined lives in a gradient between the marine and freshwater environments, the nine-spined stickleback is predominantly a freshwater inhabitant [18]. Previous studies in teleost fish have found that sensing osmotic stress by the skin cells through a transient potential receptor vanilloid 4, the differential activation or repression of several genes related to variated physiological functions could be induced [47,48]. Indeed, our study's particular relevance is that both admixture samples have been sampled in the stream's estuary, where both species coexist, and environmental changes are more pronounced due to the tidal activity. However, the three-spined (*G. aculeatus*) has a higher tolerance of salinity than the nine-spined (*P. pungitius*). Recently, a transgenerational plasticity process based on epigenetic marks and epimutations has been proposed to contribute to adaptation and acclimation of stickleback to salinity [49]. However, despite the exciting findings reported, such epigenetic studies and our genomic data interplay still maintain the salinity adaptation capacity of *G. aculeatus* unresolved. Thus, subsequent studies showing if *P. pungitius* requires the translocation of critical alleles of *G. aculeatus* to colonize into the seawater environment are guaranteed.

## 4 Conclusions

In this paper by using several independent methods, we managed to provide a hypothesis on the possibility of genomic introgression between two genera of sticklebacks (*Pungitius* and *Gasterosteus*) in northwestern Russia. Besides, a new molecular method with high power and few false positives over other related tests was provided. This convenient TE PCR marker system, developed, may contribute to fast and quality sampling of the specimens in the upcoming population-wide studies of Gasterosteiformes, with intergeneric hybridization traces. In short, if the primer system amplifies *Pungitius* specimens, the probability of *Gasterosteus* introgression would be high in that sample. We pre-accept that this method is sensitive to the group genomic introgression due to TE copies' multiplicity in the genome of animals.

## 5 Materials and methods

### 5.1 Fish sampling

Two sympatric species of the Gasterosteidae family commonly inhabiting variated niches in the Northern hemisphere, namely the three-spined (*Gasterosteus aculeatus*), and the nine-spined (*Pungitius pungitius*) stickleback were captured in the coastal lands of Chkalovsky village in the Republic of Karelia, Russia (Table 5).

**Table 5:** Nomenclature and sampling location of the three- and nine-spined stickleback samples were used on this study's variated analyses.

Sample name	Species	Sampling location∗	Sampling positions (GPS)
SH1	*Gasterosteus aculeatus*	Chkalovsky stream	66.296781, 33.398263
SH2		Chkalovsky stream	66.296781, 33.398263
S3M1		Chkalovsky village, White Sea	66.298404, 33.320415
SH3	*Pungitius pungitius*	Chkalovsky stream	66.296781, 33.398263
SH4		Chkalovsky stream,	66.296781, 33.398263
SH5		Chkalovsky stream	66.296781, 33.398263
S9C1		Quarry near Chkalovsky village	66.298542, 33.344362
S9C2		Quarry near Chkalovsky village	66.298542, 33.344362

∗ (All specimens were collected in the Republic of Karelia, Russia).

**Figure 4:**
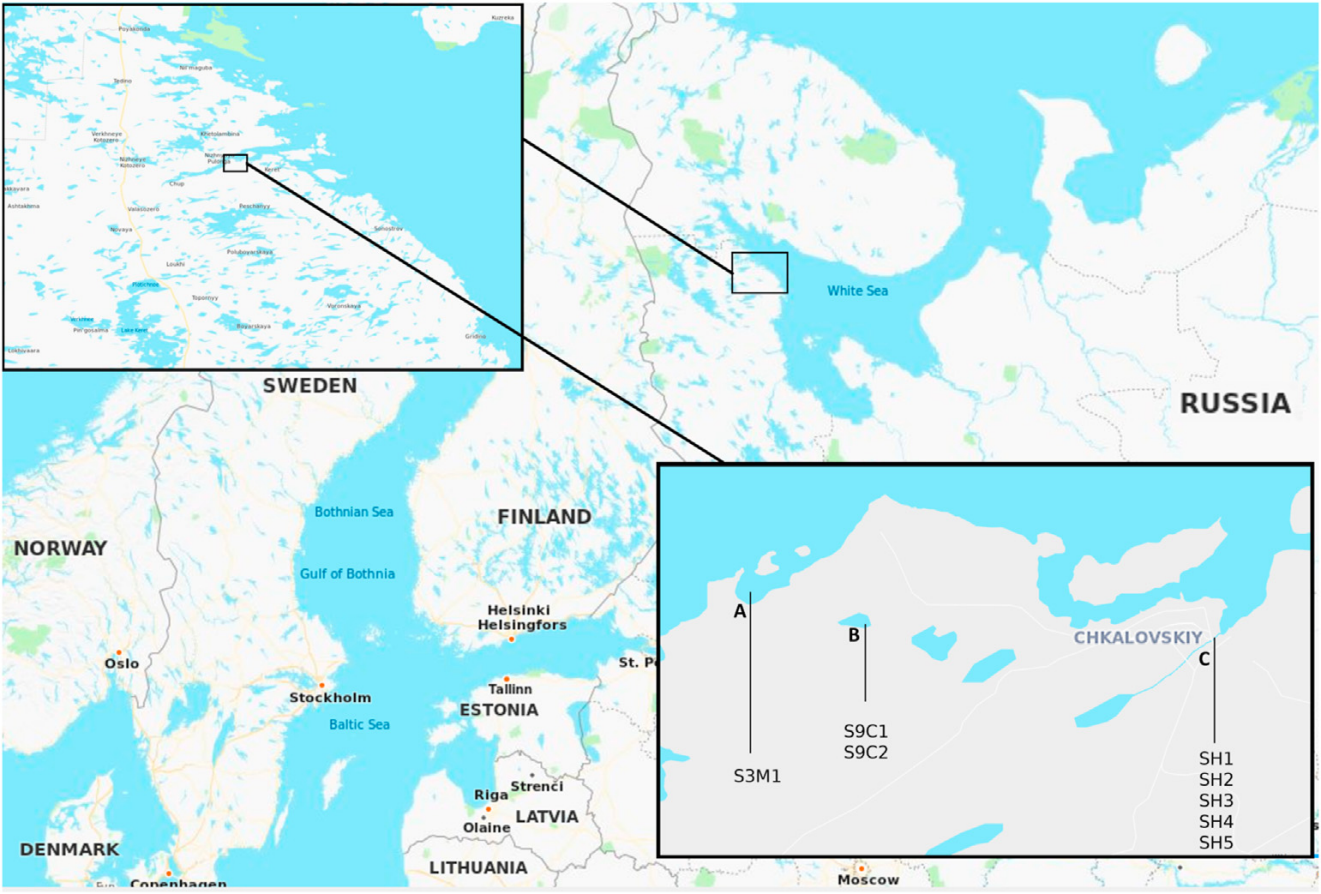
Map showing the different locations in Karelia, Russia, where *Gasterosteus aculeatus* (Three-spined) and *Pungitius pungitius* (Nine-spined) stickleback samples were collected. A) Chkalovsky village (S3M1), B) Chkalovsky quarry (S9C1 and S9C 2), C) Chkalovsky stream (SH1 to SH5) (See Table 1 for further details).

Individuals of both species were collected across a salinity gradient from the brackish White Sea waters to the freshwater in the stream within the same geographical area. Indeed, three-spined stickleback samples were collected from the White sea, near the Chkalovsky village (Figure 4-A). Samples of both species were obtained from the freshwater stream in a portion flowing into the White sea (near the tidal zone), where both species cohabitate (Figure 4-B). Nine-spined samples were collected from the freshwater quarry (Figure 4-C).

After phenotypical validation of each stickleback specimen, conducted by *in situ* dorsal fin spine counting, the anal fin was clipped and preserved in 70% ethanol for genomic DNA isolation, as described previously [50]. All specimens were caught and immediately released without any further morphological analysis to avoid stressing much the fish. One of the nine-spined sticklebacks (SH3 sample) presented a reduction in the number of spines, yet it was also used to conduct genetic analysis. This work was carried out in accordance with relevant guidelines and regulations and was approved by ethical committee of Institute of Bioengineering, Research Center of Biotechnology of the Russian Academy of Sciences, Moscow, Russia.

### 5.2 DNA extraction, and transposable element (TE) analysis

Total DNA was extracted from each stickleback sample by using the routine phenol-chloroform method. Each DNA sample concentration was quantified using a Qubit fluorimeter (Thermo Fisher Scientific, USA). The TE analysis is one of the most sensitive methods for introgression detection in animals [51]. In this work, the publicly available published sequences of the three-spine stickleback TE have been used [52]. Besides, we designed specific oligonucleotide PCR primers for amplifying variated TE fragments of several stickleback samples available. Illumina nucleotide reads of the Japanese nine-spined stickleback available at the NCBI sequence read archive (DRX012173) were used to design the specific primers for TE PCR. The Japanese nine-spined stickleback reads were mapped to the three-spined stickleback TE as the reference. Primers were designed to amplify TE's fragments that are not covered by Japanese nine-spined stickleback DNA reads. Thus, these primers could amplify only the three-spined stickleback TE but did not do so on the nine-spined ones. As a consequence, twelve TE were selected, five RNA, and seven DNA transposons, respectively. The resulting TE and the oligonucleotide primer sequences are presented (Table 6).

**Table 6:** Transposable element (TE) of *G. aculeatus* and the primer list used for their amplification.

Transposable Element		Primer (Direction)	Sequence (5' - 3′)	PCR fragment length (nt)
Class	Name			
DNA	*Ltr80*	F	AGTTCCAGGGAGCTATGCTGGGT	431
		R	CGATGCCTCACGTCCAAAGAC	
	*Tcl5*	F	GGACAACCATCTCTGCAGCAC	231
		R	GATGAGCAGTGCCTGGTTTCC	
	*Tcl7*	F	GGTGTTTCAGATTCATGCAGCGA	496
		R	GTGCTGTCCAAAACAAGCAGTGC	
	*Tcl3*	F	CCCTTTACTTTCAGTGCAGCA	296
		R	TTGCACAGTGCTCCTTGGGA	
	*hAT1*	F	CACAGATGGAGCCCCTGCTA	321
		R	CCTCCTGATGGACCAAAAGC	
	*hAT3*	F	AAGAGCAACACGGATCAGATGC	456
		R	CATTGAGAAGCCAATGGTGC	
	*hAT5*	F	ACGTTGTGTTGATGTAGCTTGGT	296
		R	CCCTCCAAAAGGCTGTTCTC	
RNA	*Gyp37_12*	F	CCTCCTAGGCTTCCGTAGTT	346
		R	AACACAGACGGTGCCTTCTG	
	*Gyp13_5*	F	CTGTGGCAGGAGCAGTCCATCTT	296
		R	CTTTGACCCGTTGGCACTCAAGG	
	*Gyp13_10*	F	GGGTGAGTTCTGGAAAACCAGC	461
		R	GCGCTCATTAGTGGCCATGAGTG	
	*Gyp9_3F*	F	ACCCAAATAGCTGTCCGCTT	260
		R	CAGTGCGTATCTTCGGGAACC	
	*Gyp9_4*	F	GTTCCCGAAGGTACGCACTG	421
		R	TGGAGAGGTACGTTAGCCCCA	

Encyclo PCR Kit (Evrogen, Russia) was used for the evaluation of the transposable element analysis. Same conditions for all loci PCR amplification were used: initial denaturation of 95 °C for 10 min followed by 35 cycles of 94 °C for 20 s, 58 °C for 30 s; 72 °C for 60 s; and final extension for 10 min at 72 °C. The PCR amplification products were separated by electrophoresis in 1% agarose gel with ethidium bromide. Universal Hood II Gel Doc System (Bio-Rad Laboratories, USA) was used for the electrophoresis visualization.

### 5.3 Library preparation, and RAD-sequencing (RAD-Seq)

The genomic DNA was digested by using two nucleases, the *EcoRI*, and *MspI* (New England Biolabs, UK). The fragmentation estimation and the size-selection were carried out using 1% agarose gel electrophoresis. DNA fragments between 350 and 450 bp were selected and extracted from the agarose gel. The obtained DNA was purified using the QIAquick Gel Extraction Kit (QIAGEN, USA). Approximately 1 μg of fragmented DNA was used for each library preparation using the NEBNext® DNA Library Prep kit (New England Biolabs, UK); DNA-libraries were multiplexed with NEBNext Multiplex Oligos for Illumina kit (New England Biolabs, UK). According to standard Illumina cluster generation and sequencing protocols, we sequenced the libraries in an Illumina 2500 platform (Illumina, USA). On this basis, 100-bp paired-end reads were generated. The data were submitted to the Sequence Read Archive under project number PRJNA529064.

### 5.4 Illumina data analysis

The DNA reads were mapped only to reference genome of *G. aculeatus* (BROAD S1, Ensemble database version 95.1) because the nine-spined stickleback genome is currently unavailable. Mapping on the three-spined stickleback genome showed a bias in the mapping efficiency between three- and nine-spined samples RAD-Seq data, that may lead to undesirable effects in the data analysis. Therefore, the European sea bass (*Dicentrarchus labrax*) genome (dicLab v1.0c) was selected as a reference and used for annotation procedures.

### 5.5 RAD-seq mapping to the reference genome

Reads were mapped to the reference genome with a bowtie2 software package [53] using a set of global mapping parameters. DNA fragments were mapped along their entire length (from beginning to end). This approach has reduced the probability of non-orthologous mapping to relatively distant reference. After obtaining the ∗.sam files, we compress them to the ∗.bam format, sort and index the alignments, using the Samtools v1.7. SNP calling was performed using samtools and bcftools packages with maximum base quality - 30 (--min-BQ parameter) [54].

In addition, the R packages: vcfR v1.8.0 [55], adegenet v2.1.1 [56], and ape v5.0 [57]were used for the subsequent genome analysis.

### 5.6 Genotype clustering

We created a distance matrix for all samples, based on the dissimilarity of their genotypes and conducted a cluster analysis using the Neighbor-Joining method by applying the “nj” function in the ape 5.0 R-package, described by Paradis et al., (2019) [57] as a modern *ad hoc* phylogenetics analysis tool.

### 5.7 Contamination test

In order to eliminate the suspicion of contamination, we determined the number of mismatches in the aligned data for each stickleback specimen. It is known that there is a number of "incorrectly" aligned sequences in any mapping data. These sequences, that show alternative nucleotides in alignment position, usually have low statistical support to characterize them as alternative alleles, because that algorithm identifies such deviations as mismatches. Moreover, DNA-library that has DNA contamination from another sample, should have a noticeably higher level of mismatches. Thus, the ratio of the number of mismatches to the total number of mapped nucleotides is an error rate, which is an indicator of contamination. To determine the error rate, we used samtools stats command [54], which estimated, besides other, mapping error rate, which is amount mismatches to total bases mapped according to cigar string information.

### 5.8 Structure software analysis

The Structure software [29] input file containing the restriction site associated DNA (RAD) genotypes was created from ∗.vcf file using the PLINK v1.9 program [58]. The console version of the Structure program was compiled and launched on the NRC “Kurchatov Institute” computer cluster. The program was run several times with different parameters, but every time the same results were obtained. The publication included the results of the launch with the following parameters: 10,000 iterations of the burning period plus 20,000 Markov chain Monte Carlo (MCMC) replicas after burning. We used admixture ancestry and correlated allele frequency models for simulations. Uniform distribution of a priori parameters, without information about the sample origin population and geographical localization. The number of clusters – two.

### 5.9 D-statistics with admixtools program suite

The D-statistics method was used to formally evaluate whether a stickleback specimen displays DNA from a distantly related population. Indeed, the next three logical steps were applied to the admixture estimation:

1) *De novo* locus building from RAD-Seq data of stickleback specimens -requires assistance form the Stacks software package v.2.53 [59]. The denovo_map.pl pipeline was used for building stacks (loci) catalog and mapping reads from each specimen to the catalog.
2) The ancestor allele state was estimated to increase the test accuracy. Estimation was conducted only for derived alleles. We mapped each stack sequence to the reference (dicLab v1.0c, PRJEB5099) genome. The nucleotide, located in the SNP site on the reference genome, was considered an ancestor allele.
3) D-statistics estimation in Admixtools software suite [30] was used. Besides, the convertf and qpDstat tools were utilized for input file conversion and D-statistics and confidence values estimation.

### 5.10 TreeMix analysis

Graph-based models were used to determine the genomic admixture (migration) between different stickleback specimens; TreeMix package [39] was utilized in this analysis. The input file was converted from a genlight (adegent R package) object using the “gl2treemix” function of the dartR [60] R package. Before converting the TreeMix file, we created the multi-vcf file with the vcfR package, filtered the loci by genotyping quality “getQUAL” function for each locus - more than 500, and removed all loci, genotypes of which were the same for all samples. Tremix was launched with the parameter defining the number of migrations equal 2. The visualization of the ancestry graph and the migration was performed using the “plot_tree” R functions of the TreeMix package.

## Declarations

### Author contribution statement

Artem Nedoluzhko, Jorge Galindo-Villegas, Sergey Rastorguev: Conceived and designed the experiments; Wrote the paper.

Artem Nedoluzhko, Fedor Sharko, Svetlana Tsygankova, Eugenia Boulygina, Amina Ibragimova, Sergey Rastorguev: Performed the experiments; Analyzed and interpreted the data.

Anton Teslyuk: Analyzed and interpreted the data.

### Funding statement

This work was supported by the 10.13039/501100002261Russian Foundation for Basic Research (#19-04-00033) and by the 10.13039/501100012190Ministry of Science and Higher Education of the Russian Federation (#075-15-2019-1659). Nord University Open Access Fund covers the OA publication costs.

### Data availability statement

Data associated with this study has been deposited at the NCBI under the accession number SRR8790922 - SRR8790929.

### Declaration of interests statement

The authors declare no conflict of interest.

### Additional information

No additional information is available for this paper.

## Appendix A Supplementary data

manuscript_stickleback_supplementary_V2 manuscript_stickleback_supplementary_V2

## References

[1] Borkin L.J., Litvinchuk S.N. (2013). Animal Hybridization, Speciation and Systematics.

[2] (1940). Speciation as a stage in evolutionary divergence. Am. Nat..

[3] Chen J., Luo M., Li S., Tao M., Ye X., Duan W., Zhang C., Qin Q., Xiao J., Liu S. (2018). A comparative study of distant hybridization in plants and animals. Sci. China Life Sci..

[4] Mallet J. (2005). Hybridization as an invasion of the genome. Trends Ecol. Evol..

[5] Abbott R., Albach D., Ansell S., Arntzen J.W., Baird S.J.E., Bierne N., Boughman J., Brelsford A., Buerkle C.A., Buggs R., Butlin R.K., Dieckmann U., Eroukhmanoff F., Grill A., Cahan S.H., Hermansen J.S., Hewitt G., Hudson A.G., Jiggins C., Jones J., Keller B., Marczewski T., Mallet J., Martinez-Rodriguez P., Möst M., Mullen S., Nichols R., Nolte A.W., Parisod C., Pfennig K., Rice A.M., Ritchie M.G., Seifert B., Smadja C.M., Stelkens R., Szymura J.M., Väinölä R., Wolf J.B.W., Zinner D. (2013). Hybridization and speciation. J. Evol. Biol..

[6] Cui R., Schumer M., Kruesi K., Walter R., Andolfatto P., Rosenthal G.G. (2013). Phylogenomics reveals extensive reticulate evolution in *Xiphophorus* fishes. Evolution.

[7] Wallis G.P., Cameron-Christie S.R., Kennedy H.L., Palmer G., Sanders T.R., Winter D.J. (2017). Interspecific hybridization causes long-term phylogenetic discordance between nuclear and mitochondrial genomes in freshwater fishes. Mol. Ecol..

[8] Lee C.E. (2002). Evolutionary genetics of invasive species. Trends Ecol. Evol..

[9] Dlugosch K.M., Parker I.M. (2008). Founding events in species invasions: genetic variation, adaptive evolution, and the role of multiple introductions. Mol. Ecol..

[10] Prentis P.J., Wilson J.R.U., Dormontt E.E., Richardson D.M., Lowe A.J. (2008). Adaptive evolution in invasive species. Trends Plant Sci..

[11] Chiba S. (2005). Appearance of morphological novelty in a hybrid zone between two species of land snail. Evolution.

[12] Nichols P., Genner M.J., van Oosterhout C., Smith A., Parsons P., Sungani H., Swanstrom J., Joyce D.A. (2015). Secondary contact seeds phenotypic novelty in cichlid fishes. Proc. Biol. Sci..

[13] Pereira R.J., Barreto F.S., Burton R.S. (2014). Ecological novelty by hybridization: experimental evidence for increased thermal tolerance by transgressive segregation in *Tigriopus californicus*. Evolution.

[14] Yoshida K., Miyagi R., Mori S., Takahashi A., Makino T., Toyoda A., Fujiyama A., Kitano J. (2016). Whole-genome sequencing reveals small genomic regions of introgression in an introduced crater lake population of threespine stickleback. Ecol. Evol..

[15] Mallet J., Besansky N., Hahn M.W. (2016). How reticulated are species?. Bioessays.

[16] Payseur B.A., Rieseberg L.H. (2016). A genomic perspective on hybridization and speciation. Mol. Ecol..

[17] Artemov A.V., Mugue N.S., Rastorguev S.M., Zhenilo S., Mazur A.M., Tsygankova S.V., Boulygina E.S., Kaplun D., Nedoluzhko A.V., Medvedeva Y.A., Prokhortchouk E.B. (2017). Genome-wide DNA methylation profiling reveals epigenetic adaptation of stickleback to marine and freshwater conditions. Mol. Biol. Evol..

[18] Rastorguev S.M., Nedoluzhko A.V., Gruzdeva N.M., Boulygina E.S., Tsygankova S.V., Oshchepkov D.Y., Mazur A.M., Prokhortchouk E.B., Skryabin K.G. (2018). Gene expression in the three-spined stickleback (*Gasterosteus aculeatus*) of marine and freshwater ecotypes. Acta Naturae.

[19] Rastorguev S.M., Nedoluzhko A.V., Sharko F.S., Boulygina E.S., Sokolov A.S., Gruzdeva N.M., Skryabin K.G., Prokhortchouk E.B. (2016). Identification of novel microRNA genes in freshwater and marine ecotypes of the three-spined stickleback (*Gasterosteus aculeatus*). Mol. Ecol. Resour..

[20] Yoshida K., Ishikawa A., Toyoda A., Shigenobu S., Fujiyama A., Kitano J. (2019). Functional divergence of a heterochromatin-binding protein during stickleback speciation. Mol. Ecol..

[21] Jones F.C., Grabherr M.G., Chan Y.F., Russell P., Mauceli E., Johnson J., Swofford R., Pirun M., Zody M.C., White S., Birney E., Searle S., Schmutz J., Grimwood J., Dickson M.C., Myers R.M., Miller C.T., Summers B.R., Knecht A.K., Brady S.D., Zhang H., Pollen A.A., Howes T., Amemiya C., Broad, Baldwin J., Bloom T., Jaffe D.B., Nicol R., Wilkinson J., Lander E.S., Di Palma F., Lindblad-Toh K., Kingsley D.M., Institute Genome Sequencing Platform & Whole Genome Assembly Team (2012). The genomic basis of adaptive evolution in threespine sticklebacks. Nature.

[22] Terekhanova N.V., Logacheva M.D., Penin A.A., Neretina T.V., Barmintseva A.E., Bazykin G.A., Kondrashov A.S., Mugue N.S. (2014). Fast evolution from precast bricks: genomics of young freshwater populations of threespine stickleback *Gasterosteus aculeatus*. PLoS Genet..

[23] Lucek K., Roy D., Bezault E., Sivasundar A., Seehausen O. (2010). Hybridization between distant lineages increases adaptive variation during a biological invasion: stickleback in Switzerland. Mol. Ecol..

[24] Rudman S.M., Schluter D. (2016). Ecological impacts of reverse speciation in threespine stickleback. Curr. Biol..

[25] Taylor E.B., Boughman J.W., Groenenboom M., Sniatynski M., Schluter D., Gow J.L. (2006). Speciation in reverse: morphological and genetic evidence of the collapse of a three-spined stickleback (*Gasterosteus aculeatus*) species pair. Mol. Ecol..

[26] Kobayashi, H. Species of stickleback, Pungitius pungitius (L.), Pungitius tymensis (Nikolsky), and Pungitius sinensis (Guichenot), with special reference to their systematic. J. Hokkaido Gakugei University.

[27] Ziuganov V.V., Gomeluk V.Y. (1985). Hybridization of two forms of ninespine stickleback, *Pungitius pungitius* and *P. platygaster*, under experimental conditions and an attempt to predict the consequences of their contact in nature. Environ. Biol. Fish..

[28] Belyayev A. (2014). Bursts of transposable elements as an evolutionary driving force. J. Evol. Biol..

[29] Falush D., Stephens M., Pritchard J.K. (2003). Inference of population structure using multilocus genotype data: linked loci and correlated allele frequencies. Genetics.

[30] Patterson N., Moorjani P., Luo Y., Mallick S., Rohland N., Zhan Y., Genschoreck T., Webster T., Reich D. (2012). Ancient admixture in human history. Genetics.

[31] Hashimoto D.T., Senhorini J.A., Foresti F., Martínez P., Porto-Foresti F. (2014). Genetic identification of F1 and post-F1 serrasalmid juvenile hybrids in Brazilian aquaculture. PloS One.

[32] Kirschbaum F., Nguyen L., Baumgartner S., Chi H.W.L., Wolfart R., Elarbani K., Eppenstein H., Korniienko Y., Guido-Böhm L., Mamonekene V., Vater M., Tiedemann R. (2016). Intragenus (*Campylomormyrus*) and intergenus hybrids in mormyrid fish: physiological and histological investigations of the electric organ ontogeny. J. Physiol. Paris.

[33] Yoshikawa H., Xu D., Ino Y., Yoshino T., Hayashida T., Wang J., Yazawa R., Yoshizaki G., Takeuchi Y. (2018). Hybrid sterility in fish caused by mitotic arrest of primordial germ cells. Genetics.

[34] Ottenburghs J. (2018). Exploring the hybrid speciation continuum in birds. Ecol. Evol..

[35] Green R.E., Krause J., Briggs A.W., Maricic T., Stenzel U., Kircher M., Patterson N., Li H., Zhai W., Fritz M.H.-Y., Hansen N.F., Durand E.Y., Malaspinas A.-S., Jensen J.D., Marques-Bonet T., Alkan C., Prüfer K., Meyer M., Burbano H.A., Good J.M., Schultz R., Aximu-Petri A., Butthof A., Höber B., Höffner B., Siegemund M., Weihmann A., Nusbaum C., Lander E.S., Russ C., Novod N., Affourtit J., Egholm M., Verna C., Rudan P., Brajkovic D., Kucan Ž., Gušic I., Doronichev V.B., Golovanova L.V., Lalueza-Fox C., de la Rasilla M., Fortea J., Rosas A., Schmitz R.W., Johnson P.L.F., Eichler E.E., Falush D., Birney E., Mullikin J.C., Slatkin M., Nielsen R., Kelso J., Lachmann M., Reich D., Pääbo S. (2010). A draft sequence of the Neandertal genome. Science.

[36] Pereira C.S.A., Aboim M.A., Ráb P., Collares-Pereira M.J. (2014). Introgressive hybridization as a promoter of genome reshuffling in natural homoploid fish hybrids (*Cyprinidae, Leuciscinae*). Heredity.

[37] Dowling T.E., Markle D.F., Tranah G.J., Carson E.W., Wagman D.W., May B.P. (2016). Introgressive hybridization and the evolution of lake-adapted catostomid fishes. PloS One.

[38] Nolte A.W. (2020). Genomic access to the diversity of fishes. Methods Mol. Biol..

[39] Pickrell J.K., Pritchard J.K. (2012). Inference of population splits and mixtures from genome-wide allele frequency data. PLoS Genet..

[40] Guerreiro M.P.G. (2014). Interspecific hybridization as a genomic stressor inducing mobilization of transposable elements in Drosophila. Mobile Genet. Elem..

[41] Díaz-Arce N., Rodríguez-Ezpeleta N. (2019). Selecting RAD-seq data analysisparameters for population genetics: the more the better?. Front. Genet..

[42] Mattucci F., Galaverni M., Lyons L.A., Alves P.C., Randi E., Velli E., Pagani L., Caniglia R. (2019). Genomic approaches to identify hybrids and estimate admixture times in European wildcat populations. Sci. Rep..

[43] Souissi A., Bonhomme F., Manchado M., Bahri-Sfar L., Gagnaire P.-A. (2018). Genomic and geographic footprints of differential introgression between two divergent fish species (*Solea* spp.). Heredity.

[44] Ford A.G.P., Dasmahapatra K.K., Rüber L., Gharbi K., Cezard T., Day J.J. (2015). High levels of interspecific gene flow in an endemic cichlid fish adaptive radiation from an extreme lake environment. Mol. Ecol..

[45] Schwenk K., Brede N., Streit B. (2008). Introduction. Extent, processes and evolutionary impact of interspecific hybridization in animals. Philos. Trans. R. Soc. Lond. B Biol. Sci..

[46] LeClere J.B., Hoaglund E.P., Scharosch J., Smith C.E., Gamble T. (2012). Two naturally occurring intergeneric hybrid snakes ( *Pituophis catenifer sayi* × *Pantherophis vulpinus;* lampropeltini, squamata) from the midwestern United States. J. Herpetol..

[47] Galindo-Villegas J., Montalban-Arques A., Liarte S., de Oliveira S., Pardo-Pastor C., Rubio-Moscardo F., Meseguer J., Valverde M.A., Mulero V. (2016). TRPV4-Mediated detection of hyposmotic stress by skin keratinocytes activates developmental immunity. J. Immunol..

[48] Bossus M., Charmantier G., Lorin-Nebel C. (2011). Transient receptor potential vanilloid 4 in the European sea bass *Dicentrarchus labrax*: a candidate protein for osmosensing. Comp. Biochem. Physiol. Mol. Integr. Physiol..

[49] Heckwolf M.J., Meyer B.S., Häsler R., Höppner M.P., Eizaguirre C., Reusch T.B.H. (2020). Two different epigenetic information channels in wild three-spined sticklebacks are involved in salinity adaptation. Sci. Adv..

[50] Kosuta C., Daniel K., Johnstone D.L., Mongeon K., Ban K., LeBlanc S., MacLeod S., Et-Tahiry K., Ekker M., MacKenzie A., Pena I. (2018). High-throughput DNA extraction and genotyping of 3dpf zebrafish larvae by fin clipping. J. Vis. Exp..

[51] Dennenmoser S., Sedlazeck F.J., Schatz M.C., Altmüller J., Zytnicki M., Nolte A.W. (2019). Genome-wide patterns of transposon proliferation in an evolutionary young hybrid fish. Mol. Ecol..

[52] Gao B., Shen D., Xue S., Chen C., Cui H., Song C. (2016). The contribution of transposable elements to size variations between four teleost genomes. Mob DNA.

[53] Langmead B., Salzberg S.L. (2012). Fast gapped-read alignment with Bowtie 2. Nat. Methods.

[54] Li H. (2011). A statistical framework for SNP calling, mutation discovery, association mapping and population genetical parameter estimation from sequencing data. Bioinformatics.

[55] Knaus B.J., Grünwald, vcfr N.J. (2017). A package to manipulate and visualize variant call format data in R. Mol. Ecol. Resour..

[56] Jombart T., Ahmed I. (2011). Adegenet 1.3-1: new tools for the analysis of genome-wide SNP data. Bioinformatics.

[57] Paradis E., Schliep K. (2019). Ape 5.0: an environment for modern phylogenetics and evolutionary analyses in R. Bioinformatics.

[58] Chang C.C., Chow C.C., Tellier L.C., Vattikuti S., Purcell S.M., Lee J.J. (2015). Second-generation PLINK: rising to the challenge of larger and richer datasets. GigaScience.

[59] Rochette N.C., Rivera-Colón A.G., Catchen J.M. (2019). Stacks 2: analytical methods for paired-end sequencing improve RADseq-based population genomics. Mol. Ecol..

[60] Gruber B., Unmack P.J., Berry O.F., Georges, dartr A. (2018). An r package to facilitate analysis of SNP data generated from reduced representation genome sequencing. Mol. Ecol. Resour..

