# Supplementary material for: Genomic evidence supports the introgression between two sympatric stickleback species inhabiting the White Sea basin
Source: Heliyon. 2021 Feb 8;7(2):e06160. doi: 10.1016/j.heliyon.2021.e06160 (PMC7875830; doi:10.1016/j.heliyon.2021.e06160)

**Supplementary Materials for**

Genomic evidence supports the introgression between two sympatric stickleback species inhabiting the White Sea basin

Artem Nedoluzhko ^1,*^, Fedor Sharko ^2,3^, Svetlana Tsygankova ^3^, Eugenia Boulygina ^3^, Amina Ibragimova ^3^, Anton Teslyuk ^3^, Jorge Galindo-Villegas ^1,*^ and Sergey Rastorguev ^3^

1. Faculty of Biosciences and Aquaculture, Nord University, 8049 Bodø, Norway.
2. Institute of Bioengineering, Research Center of Biotechnology of the Russian Academy of Sciences, 119071, Moscow, Russia.
3. National Research Center “Kurchatov Institute”, 123182 Moscow, Russia.

* Corresponding authors:

[jorge-galindo@usa.net](mailto:Jorge-galindo@usa.net); Phone +47-755-170-48

[arte](mailto:arte)m.nedoluzhko@nord.no; Phone: +47-469-013-69Figure S1. Gel electrophoresis confirming the presence of three-spined transposable elements (TE) in the nine-spined stickleback samples. To avoid overwriting each sample name and order is only indicated in the Tcl7 locus, but the same applies to each of the 12 loci presented.


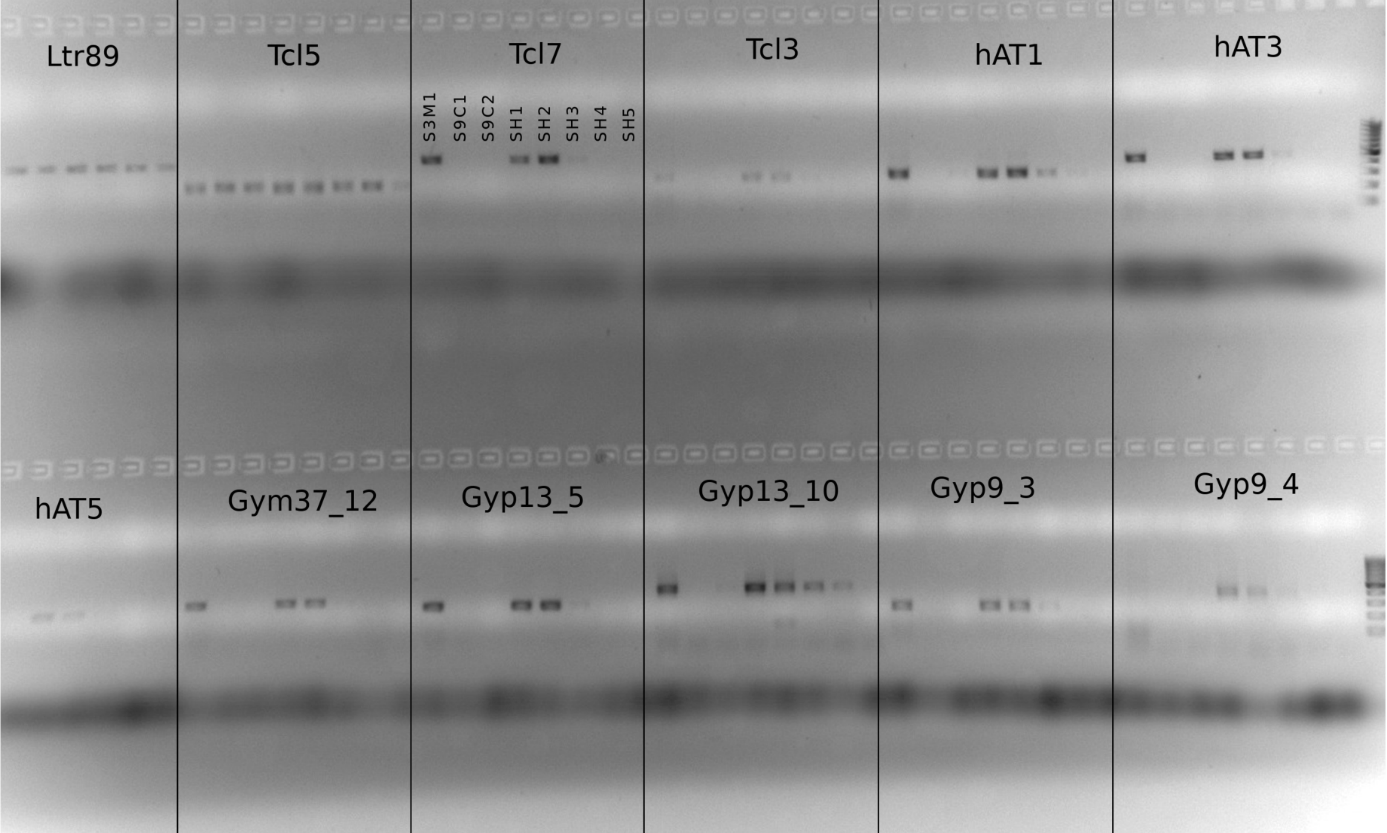

Supplement: manuscript_stickleback_supplementary_V2 [file mmc1.docx]
